# O.1.2-8 Wants to, but doesn’t do: conflict between goals and activities in community-dwelling older people

**DOI:** 10.1093/eurpub/ckad133.095

**Published:** 2023-09-11

**Authors:** Merja Rantakokko, Milla Saajanaho, Katja Kokko, Timo Törmäkangas, Erja Portegijs, Taina Rantanen

**Affiliations:** Gerontology Research Center, Faculty of Sport and Health Sciences, University of Jyväskylä, Finland; The Wellbeing Services County of Central Finland, Jyväskylä, Finland; Faculty of Education and Psychology, University of Jyväskylä, Finland; Gerontology Research Center, Faculty of Sport and Health Sciences, University of Jyväskylä, Finland; Gerontology Research Center, Faculty of Sport and Health Sciences, University of Jyväskylä, Finland; University of Groningen, University Medical Center Groningen, Center for Human Movement Sciences, Groningen, Netherlands; merja.h.rantakokko@jyu.fi; Gerontology Research Center, Faculty of Sport and Health Sciences, University of Jyväskylä, Finland

## Abstract

**Purpose:**

Active aging refers to the process where older people strive for activity in line with their goals, abilities and opportunities. However, the will does not always lead to action, creating a conflict between goals and actions. The aim of this study was to determine in which activities older people have conflicting goals and actions, and whether ability and perceived opportunity to engage in these activities explain this difference.

**Methods:**

The study included 1021 participants aged 75, 80, or 85 years, who were interviewed for the Active Ageing (AGNES) project in Jyväskylä in 2017-2018. The University of Jyvaskyla Active Aging Scale (UJACAS) includes 17 activities, and for each one, participants were asked about their desire, ability, opportunity, and frequency of participation. A conflict between goals and actions was identified if a participant expressed a desire to participate in an activity but did it rarely or not at all. Logistic regression was used for analyses.

**Results:**

The most common conflicts were related to participating in events to do with studying, clubs or associations (29%), helping others (25%), and participating in hobbies related to art and music (22%). Reduced ability to participate in art and music hobbies was associated with a conflict between goals and actions in that activity (OR 1.39, 95% CI 1.02-1.91). Perceived lack of opportunity was associated with the conflict in helping others (OR 2.04, 95% CI 1.31-3.20), when ability to engage in the activity was taken into account. Reduced ability and lack of opportunity were not associated with the conflict between goals and actions in participating in events of studying, clubs or associations.

**Conclusion:**

Ability and perceived opportunity to engage in activities only partially explain why people do not do what they want to do. Older people may prioritize their time and resources, choosing to engage in activities they consider most important. Therefore, policies and programs that promote active ageing should consider the unique needs that older people have and support access to community-based resources.

The study is funded by European Research Council and Academy of Finland.

